# Clinical and Faecal Outcomes in Adult Dogs with Chronic Enteropathy Fed an Hydrolysed Protein Diet with and Without *Bacillus velezensis* DSM 15544: A Randomised, Double-Blind, Cross-Over Trial

**DOI:** 10.3390/ani16162581

**Published:** 2026-08-18

**Authors:** Carla Giuditta Vecchiato, Federica Sportelli, Eleonora Masiero, Carlo Pinna, Martina Crisonà, Alessandra Costa, Francesca Del Baldo, Marco Giancarlo Cavazzoni, Giada Morelli, Jan S. Suchodolski, Bruna Correa Lopes, Giacomo Biagi

**Affiliations:** 1 Department of Veterinary Medical Sciences, University of Bologna, Via Tolara di Sopra 50, 40064 Ozzano Emilia, Italy; federica.sportelli4@unibo.it (F.S.); carlo.pinna2@unibo.it (C.P.); francesca.delbaldo2@unibo.it (F.D.B.); giacomo.biagi@unibo.it (G.B.); 2 Diusapet Italia S.r.l., Strada Provinciale 8 per Lardirago, 27010 Marzano, Italy; 3 Gastrointestinal Laboratory, Department of Small Animal Clinical Sciences, Texas A&M University, College Station, TX 77840, USA; jsuchodolski@cvm.tamu.edu (J.S.S.); brunalopes@tamu.edu (B.C.L.)

**Keywords:** chronic enteropathy (CE), food responsive enteropathy (FRE), hydrolysed protein, probiotics, coating, microbiota

## Abstract

Chronic enteropathy (CE) is a common cause of persistent gastrointestinal signs in dogs, and dietary management represents the cornerstone of treatment. Probiotics have been proposed as adjunctive strategies to support intestinal health, although evidence of their efficacy remains limited. This randomised, double-blind crossover trial evaluated the effects of a hydrolysed protein diet supplemented or not with *Bacillus velezensis* DSM 15544 applied during post-extrusion coating. Thirteen CE dogs were randomly assigned to receive the Diet with Probiotic (DIET+PRO) or the Diet without Probiotic (DIET) during two consecutive 60-day feeding periods, with parameters assessed at baseline (T0) and after 30 (T1) and 60 (T2) days of each dietary treatment. Clinical response was assessed using the Canine Inflammatory Bowel Disease Activity Index (CIBDAI) andfecal core (FS). Gut microbiota was evaluated by the qPCR Dysbiosis Index (DI) and 16S rRNA gene sequencing, including taxonomic composition, alpha and beta diversity analyses. Compared with baseline (CIBDAI: 4.2 ± 1.7; FS: 3.9 ± 1.5), both dietary treatments significantly improved clinical scores (CIBDAI: 1.3–1.5; FS: 2.2–2.3; *p* < 0.02), irrespective of probiotic supplementation. Neither microbiota composition, alpha diversity, beta diversity, nor the DI (−2.7 ± 2.3 to −2.1 ± 2.3) differed between dietary treatments. Clinical responses to *B. velezensis* DSM 15544 supplementation varied among dogs. Overall, the hydrolysed diet effectively improved gastrointestinal clinical signs in dogs with CE, whereas the effects of *B. velezensis* DSM 15544 remain unclear and warrant further investigation, as any additional benefit may have been masked by the marked clinical response to the hydrolysed diet.

## Simple Summary

Chronic intestinal diseases are common in dogs and often cause persistent digestive problems such as diarrhoea, vomiting, and weight loss. Dietary management is usually the first treatment approach, and hydrolysed protein diets are widely used because they reduce the risk of adverse reactions to food proteins. In recent years, probiotics—live microorganisms that may support gut health—have been proposed as an additional nutritional strategy, although their effectiveness in dogs with chronic intestinal disease is still uncertain. This study evaluated whether adding the probiotic bacterium *Bacillus velezensis* to the kibbles’ coating of a hydrolysed protein diet could provide additional benefits in dogs with chronic enteropathy. Thirteen dogs were enrolled in a randomised, double-blind crossover trial and received the diet with and without the probiotic. Overall, the hydrolysed diet improved gastrointestinal signs and stool quality in most dogs. However, no statistically significant differences between the two groups emerged, and the effects of *B. velezensis* remain unclear and warrant further investigation.

## 1. Introduction

Chronic enteropathies (CEs) are among the most common diseases in dogs, characterised by persistent intestinal inflammation associated with chronic or recurrent gastrointestinal (GI) signs [1]. These include diarrhoea, vomiting, reduced appetite, weight loss, borborygmi, flatulence, eructation, and abdominal pain, which can occur independently or concurrently [2,3]. CE are considered multifactorial conditions resulting from complicated interactions between genetic susceptibility, immune dysregulation, environmental factors, imbalances of the intestinal microbiota, and dietary components. Among the different forms of canine CE, a substantial proportion of cases respond to dietary modification alone and are therefore classified as food-responsive enteropathies (FREs) [1,4,5]. Previous studies have reported that up to 70% of dogs with chronic enteropathy experience clinical improvement following dietary intervention [1,6,7]. For this reason, dietary interventions based on novel or hydrolysed protein sources are commonly used as first-line therapeutic strategies [4,8,9]. More specifically, hydrolysed protein diets contain proteins that are enzymatically cleaved into low-molecular weight peptides, thereby reducing antigenicity and limiting adverse immune reactions associated with the intestinal mucosa [10].

Beyond dietary management, increasing attention has been directed towards approaches aimed at modulating the intestinal microbiota. In fact, growing evidence indicates that dogs affected by CE often exhibit alterations in gut microbial composition, commonly referred to as dysbiosis, characterised by reduced microbial diversity and shifts in the abundance of key bacterial taxa [11]. Consequently, interventions like prebiotics, probiotics, postbiotics, and faecal microbiota transplantation are being progressively investigated as adjunctive therapeutic approaches [12].

Among these strategies, probiotics have attracted considerable interest as potential antibiotic-sparing tools capable of modulating both the intestinal ecosystem and host immune responses [13]. Probiotics are defined as live microorganisms that, when administered in adequate amounts, confer a health benefit on the host [14]. Their mechanism of action is multifactorial and includes production of antimicrobial compounds and organic acids, competitive exclusion of pathogens, and reinforcement of intestinal barrier integrity [15,16]. In addition, experimental studies have shown that probiotics may stimulate mucosal immunity through interactions with gut-associated lymphoid tissue (GALT), promoting immunoglobulin A (IgA) secretion and modulating cytokine production [17,18].

Despite the promising mechanisms, clinical evidence supporting the efficacy of probiotics in canine CE remains limited and strongly strain-dependent [19,20,21,22]. The viability and stability of probiotic microorganisms are key determinants of their efficacy. Their incorporation directly into pet food formulations may facilitate daily administration and improve owner compliance [16]; however, the high temperatures and mechanical stress associated with the extrusion process may significantly reduce microbial survival. In recent years, several technological strategies have therefore been proposed to preserve viability during feed processing, including the application of probiotics during the coating phase after extrusion [23].

Indeed, previous investigations evaluating commercial pet foods labelled as containing probiotics have frequently reported discrepancies between declared and detected microbial content [16,24]. These findings underline the importance of ensuring adequate probiotic stability and viability in pet food products.

The probiotic candidate *Bacillus velezensis* DSM 15544 (formerly *B. subtilis* C-3102) has emerged as a promising microorganism in companion animal nutrition, having been associated with improvements in faecal quality and indicators of gut health in dogs [25,26,27,28], although not all studies have confirmed these benefits using another strain of *B. subtilis* [29,30]. This strain is currently authorised by the European Union for use as a feed additive in dogs and is characterised by its ability to form highly resistant spores, which ensure stability during feed processing as well as during gastrointestinal transit [31].

The present study was designed to evaluate the efficacy of a hydrolysed protein diet fortified or not with *B. velezensis* DSM 15544, administered as a coated probiotic. Specifically, in a randomised, double-blind, cross-over trial, we aimed to determine whether the inclusion of the probiotic would provide additional clinical and microbial benefits compared with the hydrolysed diet alone. Outcomes of interest included changes in clinical parameters as well as modifications in faecal microbiota composition and faecal metabolite profiles.

## 2. Materials and Methods

The research was conducted in accordance with Italian legislation transposing the European Council Directive 2010/63 on the protection of animals used for scientific purposes. The study design was approved by the University of Bologna Animal Research Ethics Committee (protocol no. 11677/2023). All dogs were recruited through the Gastroenterology Unit of the Veterinary Teaching Hospital at the University of Bologna between November 2023 and March 2024. Informed consent was obtained from each dog’s owner prior to enrolment.

### 2.1. Animals

Privately owned adult (>1 year of age) dogs presenting with chronic (≥three weeks duration) GI signs were considered for enrolment in this study. Clinical signs included diarrhoea, vomiting and/or nausea, abdominal bloating and/or pain, either alone or in combination. To be eligible, dogs must not have been previously fed any veterinary therapeutic diets based on hydrolysed protein sources formulated for diet elimination trials. All dogs remained in their usual home environment with their owners throughout the study, and no changes in housing or management were introduced by the study protocol.

At the time of enrolment (T0), all dogs underwent a comprehensive diagnostic work-up to exclude gastrointestinal or extra-gastrointestinal diseases other than chronic enteropathy. This evaluation included blood testing (complete blood count, CBC; routine serum biochemistry, including C-reactive protein measurement; basal cortisol, folate and cobalamin concentrations), urinalysis with urine protein-to-creatinine ratio (UPC), and abdominal ultrasonography. Moreover, parasitic infections had to be excluded by faecal flotation performed on pooled samples collected over three consecutive days. Dogs were excluded if abnormalities in any of the above-mentioned diagnostic tests, which could have compromised the presumptive diagnosis of idiopathic chronic enteropathy, were detected. Additional exclusion criteria included treatment with antibiotics, corticosteroids, or probiotics within the 60 days preceding enrolment, as well as the use of gastrointestinal medications (e.g., omeprazole) or supplements intended to support intestinal function during the same period. Dogs were withdrawn from the study in the event of owner non-compliance with the prescribed dietary regimen or the occurrence of severe clinicopathological alterations.

### 2.2. Diet and Study Design

This study followed a prospective, randomised, double-blind, cross-over design. At T0, dogs were randomly allocated to one of two treatment sequences: DIET or DIET+PRO. The two experimental hydrolysed diets had identical formulations (Table 1; Alleva Care Enterohypo, Diusapet S.r.l., Marzano, Pavia, Italy) and differed solely in the inclusion of *Bacillus velezensis* DSM 15544 in DIET+PRO.

*B. velezensis* DSM 15544 was incorporated into the complete feed at a target concentration of 1 × 10^9^ CFU/kg feed, as recommended by the producer. The recovery of the probiotic was performed at LTZ Augustenberg (Karlsruhe, Germany) according to the DIN EN 15784:2022-02 method [32]. The assay has an analytical uncertainty of 60% when applied to complete feed matrices. Recovery analysis showed a mean concentration of 0.825 × 10^9^ ± 0.319 × 10^9^ CFU/kg. Stability testing conducted throughout the study period confirmed that the probiotic maintained viability for the entire duration of the study.

The two diets were packed in identical white plastic bags and labelled as A or Z at the production facility (Diusapet S.r.l., Marzano, Pavia, Italy); the correspondence between letter codes and treatments was known only to the head of production and was sealed in an envelope until study completion, ensuring blinding of all investigators and personnel involved in clinical evaluations and data collection throughout the study. For each dog, the treatment sequence was randomly assigned using dedicated software (Research Randomizer, http://www.randomizer.org/) at the time of the first clinical visit, without stratification by covariates.

Before starting the study, dogs underwent a 7-day transition period during which their previous diet was gradually replaced by the first study diet (DIET or DIET+PRO, according to the assigned sequence). One group received the DIET for 60 days and was subsequently transitioned to the DIET+PRO for an additional 60 days, whereas the other group followed the opposite sequence, starting with the DIET+PRO for 60 days and then switching to the DIET for the same duration. No washout period was included between the two study phases, resulting in a total study duration of 120 days.

The daily ration of each experimental diet was calculated based on the individual dog’s ideal body weight, using the equations for adult dogs proposed by the FEDIAF nutritional guidelines [32].

### 2.3. Clinical Evaluations and Faecal Sample Collection

At T0 and after 30 days (T1) and 60 days (T2) of each dietary treatment, Body Weight (BW), Body Condition Score (BCS; 9-point scale [33]), Canine IBD Activity Index (CIBDAI [34]) and faecal score (FS; 7-point Purina Faecal Scoring Chart) were recorded. At T1 and T2 of each dietary treatment, a freshly voided faecal sample was collected and frozen within 20 min after evacuation, stored at −80 °C until chemical and microbiological analyses were performed.

### 2.4. Chemical Analyses

Blood samples were collected after 12 h of fasting; CBC was performed using an automated haematology analyser (ADVIA 2120, Siemens Healthcare Diagnostics, Tarrytown, NY, USA), while serum biochemical analyses were conducted using an automated chemistry analyser (AU480, Beckman Coulter/Olympus, Brea, CA, USA).

Proximate analyses of DIET and DIET+PRO were conducted according to International standard methods (AOAC, 2000) [35].

Faecal pH was measured after diluting the samples with deionised water at a 1:10 (*w*/*v*) ratio using a laboratory pH meter (SevenMulti, Mettler Toledo, Greifensee, Switzerland; accuracy ± 0.01), while faecal ammonia concentration was determined using an enzymatic colorimetric assay (Urea/BUN-Color; BioSystems S.A., Barcelona, Spain). Faecal volatile fatty acids (VFAs), including acetate, propionate, butyrate, isopropionate, and isobutyrate, were quantified by gas chromatography according to the method described by Pinna et al. [36]. Faecal IgA concentrations were quantified using an ELISA kit (E44-104, Bethyl Laboratories Inc., Montgomery, TX, USA).

### 2.5. Microbial Analyses

Fecal DNA extraction was performed on 100 mg (wet weight) of each faecal sample using the MoBio Power Soil DNA Isolation Kit (MO BIO Laboratories Inc., Carlsbad, CA, USA), following the manufacturer’s instructions.

The Dysbiosis Index (DI) was determined using a validated quantitative PCR (qPCR)–based assay as previously described by AlShawaqfeh et al. [11]. This method quantified core intestinal bacteria (i.e., *Peptacetobacter hiranonis*, *Faecalibacterium*, *Turicibacter*, *Streptococcus*, *Escherichia coli*, *Blautia*, *Fusobacterium*, and total bacteria), expressed as log DNA/g of faeces, which were integrated into a composite index. According to laboratory reference ranges, DI values < 0 are within the normal range, 0–2 mild dysbiosis, and DI > 2 marked or significant dysbiosis.

In addition, for 16S rRNA gene sequencing, a single-step 30-cycle PCR using the HotStarTaq Plus Master Mix Kit (Qiagen, Venlo, The Netherlands) was performed under the following conditions: 94 °C for 3 min, followed by 28 cycles (5 cycles used on PCR products) of 94 °C for 30 s, 53 °C for 40 s and 72 °C for 1 min, after which a final elongation step at 72 °C for 5 min was performed. Illumina sequencing of V4 region of the bacterial 16S rRNA genes was performed using primers 515F (5′-GTGCCAGCMGCCGCGGTAA-3′) and 806R (5′-GGACTACVSGGGTATCTAAT-3’) at the MR DNA laboratory (www.mrdnalab.com, Shallowater, Texas).

### 2.6. Statistical Analysis

Continuous variables were assessed for normality using the Shapiro–Wilk test. Data are presented as mean ± standard deviation (SD) or median and range (minimum and maximum values), depending on whether they were normally or non-normally distributed, respectively. Differences between variables were evaluated using a generalised linear model (GLM) fitting a cross-over design, including diet (DIET vs. DIET+PRO) and treatment sequence (DIET-DIET+PRO vs. DIET+PRO-DIET) as fixed factors, and subjects as random factors. The effects of period and the diet × period interaction were tested to control the possibility of carry-over effect. Post-hoc analysis within the GLM framework was conducted using pairwise tests.

Comparisons of clinicopathological variables between the two experimental diets at the different time points (T0; T1 and T2 of DIET; T1 and T2 of DIET+PRO) were performed using the Wilcoxon Rank-Sum test or paired T-test, as appropriate based on data distribution.

The demultiplexed paired-end sequences were visually inspected for quality using QIIME2’s interactive sequence quality plots (Version 2024.10). Based on the quality profiles, both forward and reverse reads were truncated at 200 bp, where average Phred scores consistently exceeded 30. Quality filtering and denoising were performed using DADA2 with default parameters, including the removal of chimeric sequences. Samples with fewer than 100,000 reads after filtering were excluded from further analysis; this cutoff ensured sufficient sequencing depth and minimised potential biases from low-coverage samples. Non-bacterial ASVs, including mitochondrial and chloroplast reads, were filtered out. For taxonomic assignments, the SILVA 138 database classifier was used with the VSEARCH algorithm. Sequence alignments were performed using MAFFT, and a phylogenetic tree was built using FastTree (built-in version available in QIIME 2 version 2024-10). For diversity analysis, feature tables were rarefied to 135,900 reads, the lowest sample read count.

Alpha diversity was calculated using the Chao1, Observed Features, and Shannon metrics to estimate community richness and evenness. A linear mixed-effects model was used to test differences in alpha diversity between groups. Beta diversity was calculated using Bray-Curtis, unweighted Unifrac, and weighted Unifrac dissimilarity matrices to estimate differences in overall community structure. Pairwise permutational multivariate analysis of variance (PERMANOVA) was calculated for all beta diversity measures using 999 permutations. *p*-values were corrected for multiple comparisons using the Benjamini–Hochberg procedure.

To assess differences in the abundance of identified bacteria at each taxonomic level, both prevalence- and abundance-based Multivariable Association with Linear Models 3 (MaAsLin3) was applied. The Benjamini–Hochberg Procedure was used globally to adjust for multiple comparisons, and a q-value < 0.05 was defined as being statistically significant.

For all statistical analyses, significance was set at *p* < 0.05. Statistical analyses were conducted using GraphPad Prism version 9.5.1 (GraphPad Software, USA) or R software version 4.4.0.

## 3. Results

### 3.1. Dogs Characteristics

Thirteen dogs met the inclusion criteria; 12/13 completed the trial, with complete datasets available for analysis. None of the dogs refused the diets, and no palatability issues were reported to the investigators. One dog was withdrawn before reaching T2 of the DIET treatment, due to reasons unrelated to the protocol. The study population comprised eight males (six intact and two neutered) and five females (one intact and four spayed). The median age was 59 months (range: 11–132 months), and the median body weight was 17 kg (1.7–36.9 kg). Three dogs were mixed breed, and ten were purebred (Labrador Retriever, *n* = 2; Poodle, *n* = 2; Chihuahua, *n* = 1; English Setter, *n* = 1; German Shepherd Dog, *n* = 1; Golden Retriever, *n* = 1; Maltese, *n* = 1; Welsh Corgi Pembroke, *n* = 1).

In accordance with the inclusion criteria, none of the enrolled dogs had previously received a hydrolysed protein diet. At enrolment (T0), dogs were being fed novel protein commercial diets (*n* = 6), novel protein home-made diets (*n* = 4), gastrointestinal commercial diets (*n* = 2), or an adult maintenance diet (*n* = 1).

At T0, serum cobalamin concentrations were defined as subnormal (<400 ng/L) in 8/13 (62%) dogs; therefore, these dogs received cobalamin supplementation throughout the study period, administered either orally (*n* = 4, 250–2000 µg q24h depending on the dog size) or with subcutaneous injections (*n* = 4, 250–1500 µg depending on the dog size), following the protocol described in [37].

**Figure 1:**
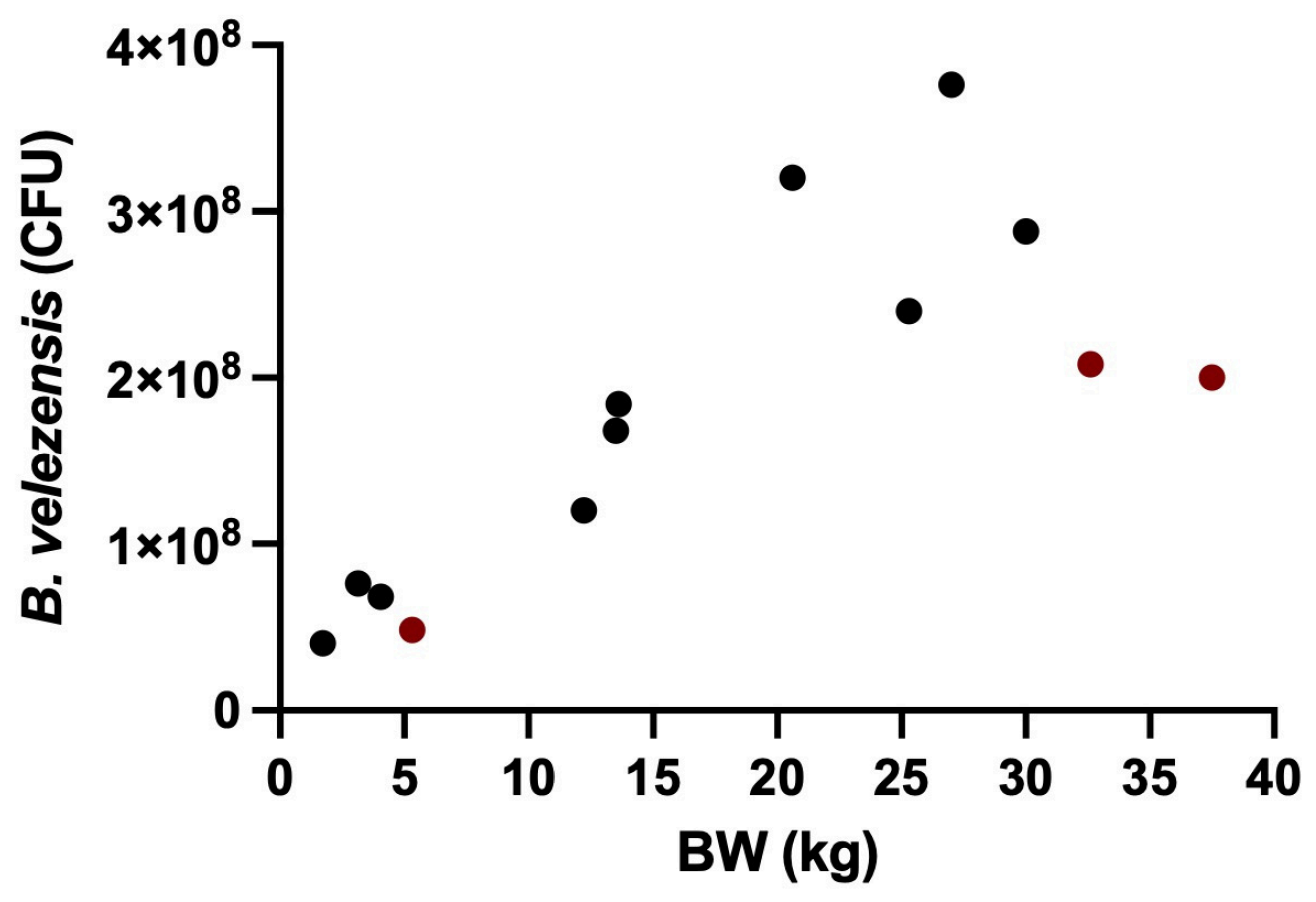
*Bacillus velezensis* consumption (CFU) in the study dogs (*n* = 13) according to their body weight (BW).

At T0, the mean daily energy intake, measured in kilocalories of metabolizable energy, was 109 ± 32 kcal/kg^0.75^, which led to the average consumption of 1.8 × 10^8^ ± 1.07 × 10^8^ CFU (range: 4.0 × 10^7^–3.8 × 10^8^ CFU) of *B. velezensis*. Because three dogs presented a baseline BCS of 7/9, they were fed a calorie-restricted diet (66 ± 5 kcal/kg metabolic body weight) to promote weight loss, whereas dogs with a normal BCS were fed at maintenance. As a result, probiotic consumption did not follow a linear pattern during the study (Figure 1, red dots).

### 3.2. Clinical Observations

Except for the three dogs fed with caloric restriction, the body weight remained largely stable over time, irrespective of diet, with a slight increase in BCS recorded at the end of the DIET+PRO period (*p* = 0.05, Table 2).

At T0, the most commonly owner-reported clinical signs were decreased faecal consistency (10/13) and increased defecation frequency (10/13), followed by reduced appetite (4/13), vomiting (4/13), and unintentional weight loss (4/13); no owner reported reduced activity levels. Already by the end of the first dietary intervention (T2, both DIET and DIET+PRO), clinical signs improved: increased defecation frequency (8/13; −20%), decreased faecal consistency (3/13; −70%), unintentional weight loss (1/10; −75%), reduced appetite (3/13; −25%), and vomiting (0/13; −100%). At the end of the study (i.e., 120 days after the start of the trial), the prevalence of most clinical signs further decreased, including increased defecation frequency (5/13; −37%), decreased faecal consistency (2/13; −33%), and reduced appetite (1/13; −67%), while unintentional weight loss and vomiting occurred in 2/10 and 1/13, respectively. Activity levels remained unchanged in all dogs.

**Figure 2:**
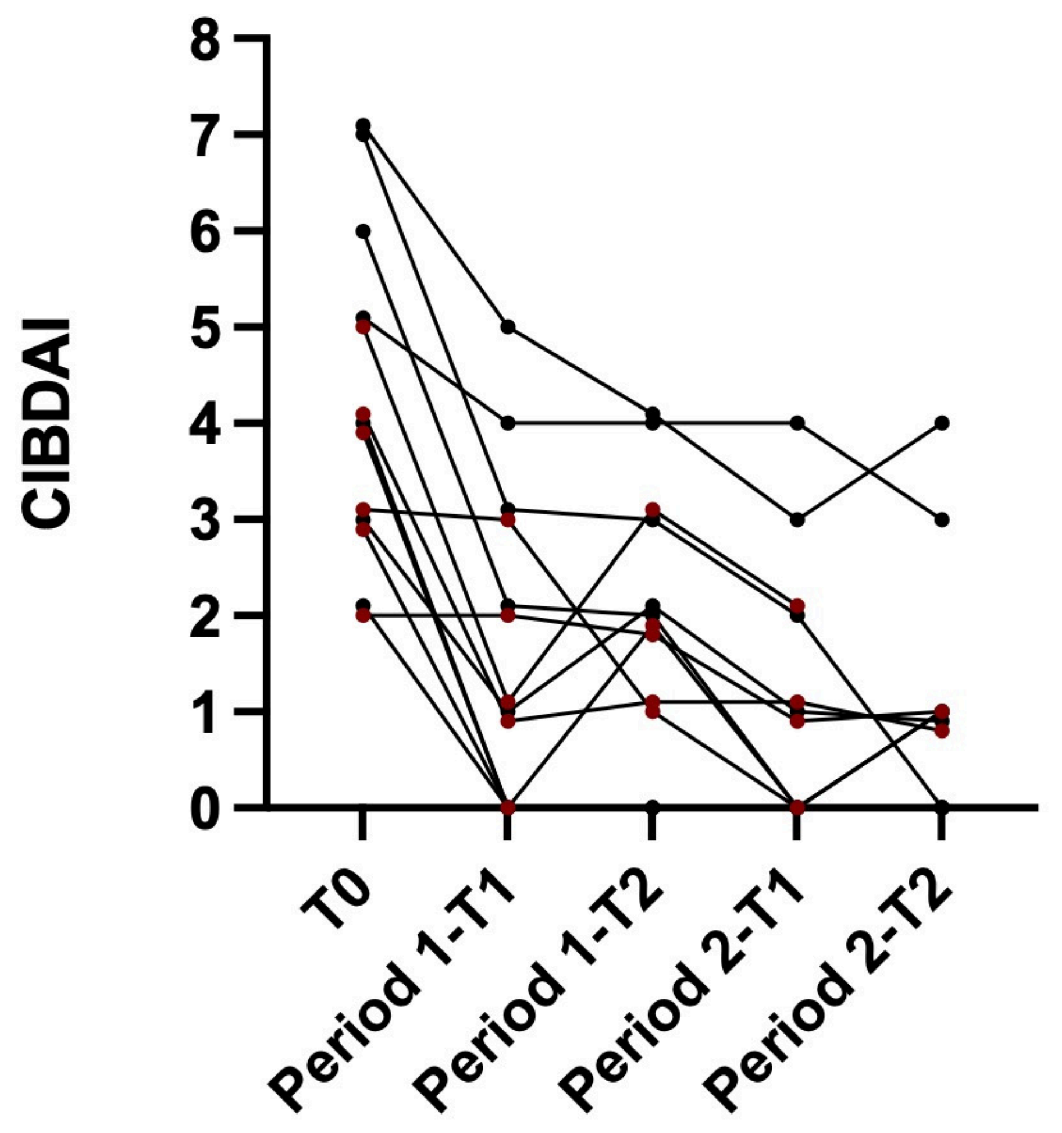
Changes in Canine Inflammatory Bowel Disease Activity Index (CIBDAI) over time in the study dogs. T0 indicates baseline; T1 and T2 indicate the sampling time points (30 and 60 days) within each of the two crossover periods (Period 1 and Period 2, respectively). Data are available for 13 dogs at T0, Period 1—T1, Period 1—T2, and Period 2—T1, and for 12 dogs at Period 2—T2, as one dog did not complete the study. Red dots represent dogs with DIET+PRO–DIET sequence; black dots represent dogs with DIET–DIET+PRO sequence.

At baseline, CIBDAI scores did not differ between dogs fed the two dietary intervention sequences (*p* = 0.144). Compared to T0, improvement in CIBDAI was observed in 11 out of 13 dogs. Specifically, improvement occurred in all dogs starting with DIET (7/7, median (range) improvement in CIBDAI score: 67% (20–100%)), and in four out of six dogs starting with DIET+PRO (median (range) improvement in CIBDAI score: 67% (0–100%). CIBDAI scores (mean ± SD) decreased significantly over the course of the study relative to T0, whereas no significant differences were detected between the two dietary treatments. Compared with baseline (T0: 4.2 ± 1.7), mean CIBDAI score during DIET+PRO was 1.3 ± 1.4 (*p* < 0.001) at T1 and 1.4 ± 1.3 (*p* < 0.002) at T2. Similarly, during DIET, the score was 1.5 ± 1.7 at T1 (*p* < 0.0001) and 1.5 ± 1.5 at T2 (*p* < 0.0001). Among the seven dogs receiving DIET followed by DIET+PRO (Figure 2, black dots), all improved at T1 compared with T0, with five reaching a clinical score between 0 and 3; between T1 and T2, five dogs remained clinically stable, one further improved, and one worsened. Following the switch to DIET+PRO, four dogs improved while three remained stable (two with a score of 0 and one with a score of 4); between T1 and T2 of the DIET+PRO period, two dogs improved, three remained stable (two with a score of 0 and one with a score of 1), and two showed a slight worsening (0 to 1 and 3 to 4, respectively).

In the reverse sequence (Figure 2, red dots), starting with DIET+PRO, 4/6 dogs improved at T1 compared with T0, all reaching a clinical score between 0 and 3, whereas two remained stable (scores of 2 and 3); between T1 and T2, three dogs remained stable, two worsened, and one improved, all remaining within the 0–3 score range. After switching to DIET, four dogs improved and two remained stable (scores of 0 and 1); at T2, one dog was withdrawn, while the remaining dogs had scores between 0 and 1, with one changing from 0 to 1.

**Figure 3:**
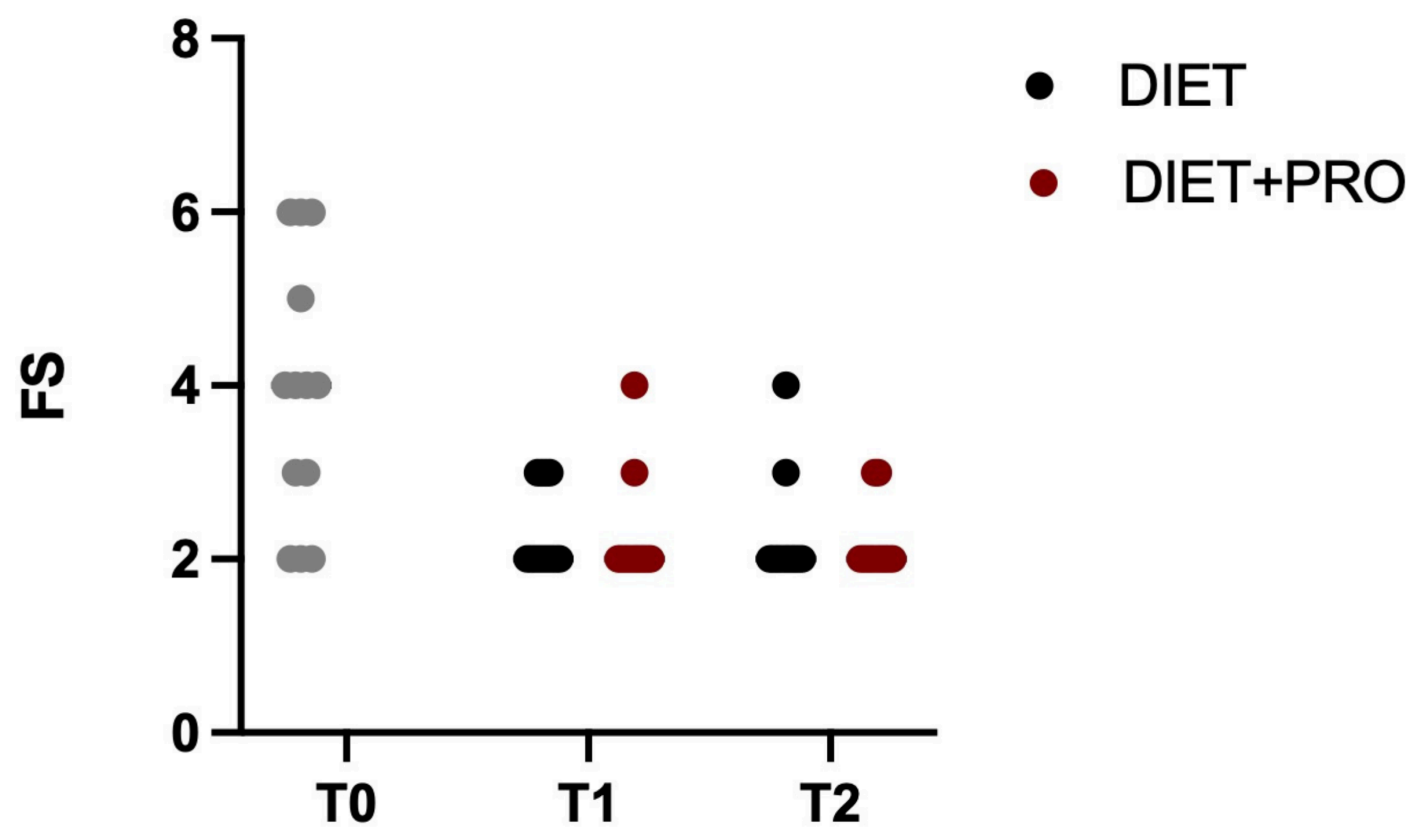
Faecal scores (FS) in the study dogs (*n* = 13) at baseline (T0, grey dots) and during the two dietary interventions at each sampling time point (T1 and T2). Data are available for 13 dogs at T0 and T1, and for 12 dogs at T2, as one dog did not complete the study. Individual dots represent single dogs.

FS at T0 did not differ between dogs fed the two dietary intervention sequences (*p* = 0.38). FS improved throughout the study, with no significant differences between the two dietary treatments (Figure 3). Compared with baseline (T0: 3.9 ± 1.5), FS values during DIET+PRO (red dots) decreased to 2.3 ± 0.5 at T1 (*p* = 0.015) and 2.2 ± 0.6 at T2 (*p* = 0.006). A comparable improvement was observed during DIET (black dots), with FS values of 2.2 ± 0.6 at T1 (*p* = 0.006) and 2.2 ± 0.4 at T2 (*p* = 0.02).

Among the 13 dogs enrolled, 5 (38%) showed a positive clinical outcome with no differences between the two diets. The remaining eight dogs (62%) exhibited different clinical responses to DIET versus DIET+PRO. In seven of these eight dogs, clinical signs worsened during DIET+PRO compared with DIET, including ocular manifestations (epiphora, conjunctival hyperaemia, periocular staining) in three dogs (23%), occasional gastrointestinal signs (diarrhoea, flatulence, reduced appetite) in three dogs (23%), and a combination of ocular signs, diarrhoea, and pruritus in one dog (8%). Only one of these eight dogs showed a more favourable clinical response during DIET+PRO compared with DIET, characterised by reduced stool mucus and decreased flatulence.

### 3.3. Faecal Metabolites and Microbiota

Faecal pH, NH_3_, IgA and VFA concentrations (expressed as % of total VFA) did not differ significantly between treatments and over time (*p* > 0.05, Table 3).

**Figure 4:**
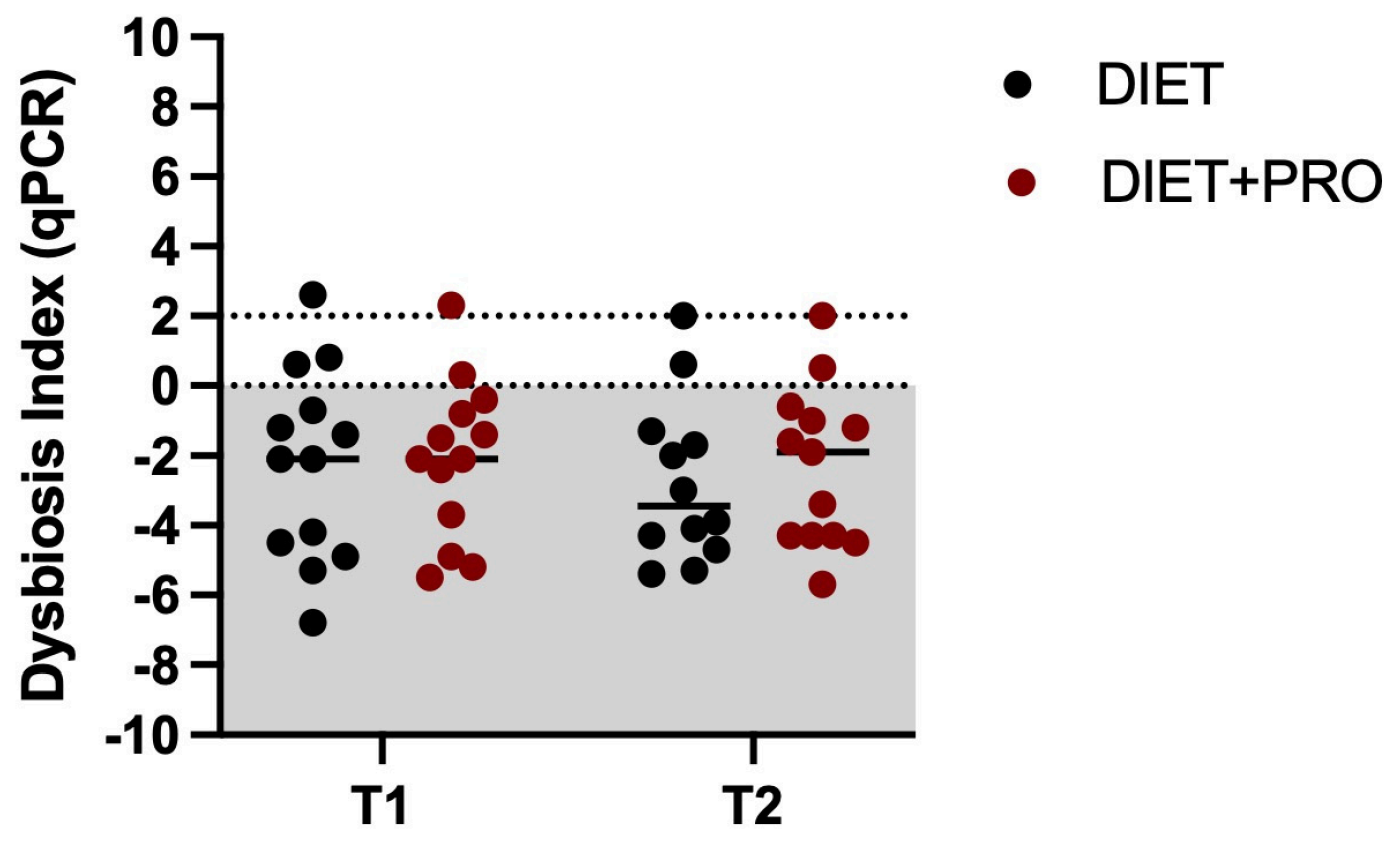
Faecal qPCR-based Dysbiosis Index (DI) of the study dogs (*n* = 13) during the two dietary interventions (DIET and DIET+PRO) at each sampling time point (T1 and T2). Interpretation of DI: DI < 0 is normal (dotted line); DI ranging from 0 to 2 is mildly increased; and DI > 2 is significantly increased. Data are available for 13 dogs at T1, and for 12 dogs at T2, as one dog did not complete the study. Individual dots represent single dogs, and the horizontal line indicates the median value.

The DI did not differ significantly between DIET+PRO and DIET at either time point (mean ± SD qPCR DI T1: DIET −2.2 ± 2.8 vs. DIET+PRO −2.1 ± 2.3; T2: DIET −2.7 ± 2.3 vs. DIET+PRO −2.3 ± 2.3, Figure 4). During the two-month consumption of each diet, most dogs (*n* = 10/13) consistently exhibited a normal DI (DI < 0), regardless of diet and sampling time (Figure 4). All but one dog in this study had DI and core bacterial populations tested by qPCR within the normal range, which remained unaffected throughout the study. Only one dog had an increased DI (DI > 2) that remained unchanged throughout the study (*p* > 0.05). In this dog, the increased DI was a consequence of a persistent deficiency of the bile acid-converting bacterium *P. hiranonis* (normal abundance: 5.1–7.1 log DNA) [11], which ranged from 0.8 to 1.6 log DNA over the study (*p* > 0.05) and did not recover as a result of the dietary treatments.

No significant changes were observed in alpha-diversity (Chao1, Observed Features, and Shannon entropy metrics) across time points, with the inclusion of the probiotic, or in the interaction between time and diet with or without the inclusion of the probiotic.

**Figure 5:**
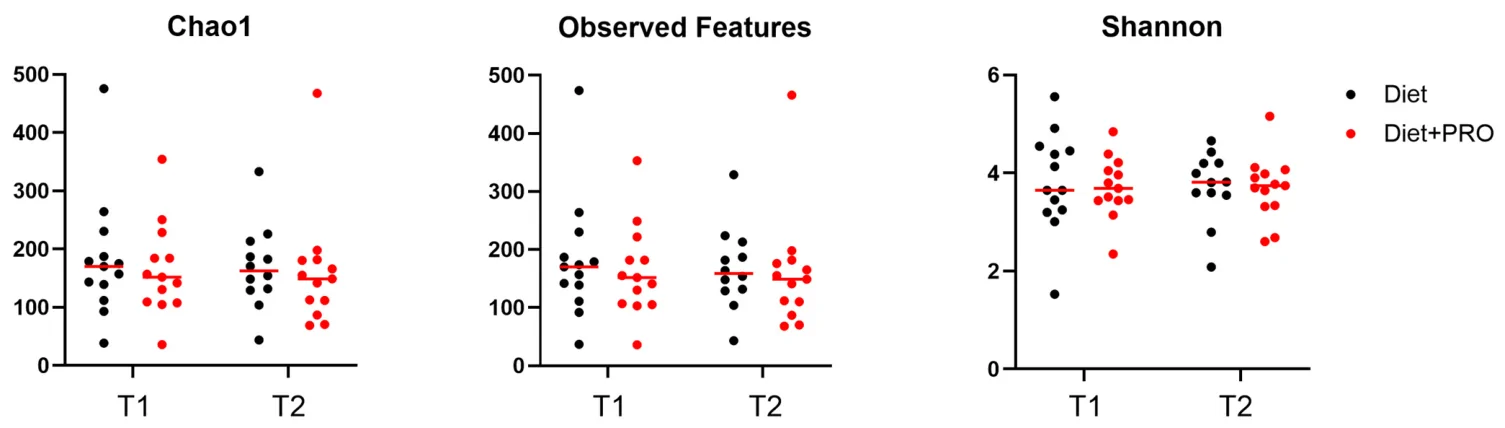
Alpha diversity was not significantly changed by diet or time. From left to right: Chao1 richness, Observed features, and Shannon entropy metrics were compared across time points and diet groups. No significant effects of time, diet, or the time × diet interaction were detected for any alpha diversity metric based on linear mixed models. Each point represents an individual sample, and boxplots show the median and interquartile range. Data are available for 13 dogs at T0 and T1, and for 12 dogs at T2, as one dog did not complete the study. Individual dots represent single dogs, and the horizontal line indicates the median value.

There was considerable between-animal variability, with no variation attributable to sex (Figure 5 and Supplementary File S1).

**Figure 6:**
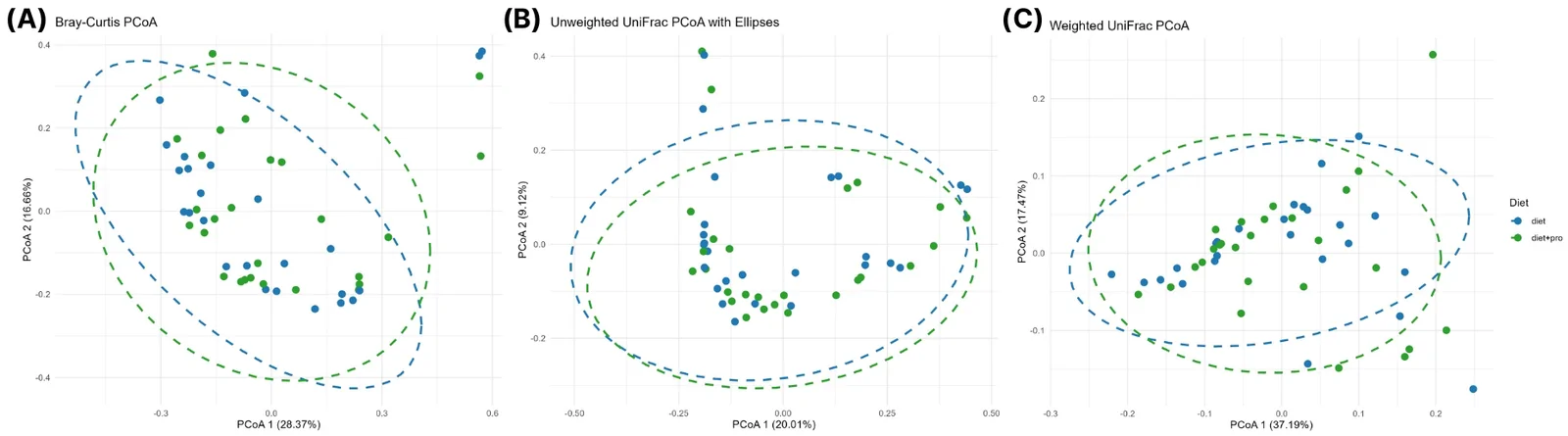
PCoA representing Bray-Curtis (**A**), unweighted UniFrac (**B**), or weighted UniFrac (**C**) distances among DIET (blue dots) and DIET+PRO (green dots). ANOSIM analysis of beta diversity showed no significant separation among groups. R values were close to zero and all *p*-values were >0.05, indicating no detectable differences in microbial community composition among groups.

For beta-diversity, the analysis showed no significant differences in microbial community composition among groups for Bray-Curtis (R = −0.031, *p* = 0.819), unweighted UniFrac (R = 0.021, *p* = 0.285), or weighted UniFrac (R = −0.043, *p* = 0.903). These findings indicate that overall microbial community structure did not differ significantly between groups (Figure 6, Supplementary File S1, Supplementary Figures S1 and S2).

Taxonomic analyses were subsequently performed to assess whether the dietary treatments differentially affected the relative prevalence and abundance of specific bacterial taxa. The main results from the MaAsLin3 model are shown in Table 4 and the Supplementary File.

The DIET+PRO treatment was associated with changes in several bacterial taxa. At the phylum level, Actinobacteriota abundance and Firmicutes prevalence were higher during the DIET+PRO period than during the DIET period. At the genus level, the prevalence of *Fournierella* and the abundance of *Lactobacillus* were lower, whereas the prevalence of *Bacillus*, and the abundances of *Eggerthella* and *Romboutsia* were higher during the DIET+PRO period than during the DIET period; however, all these results were not significant when adjusted for multiple comparisons.

In a separate analysis, the presence of adverse effects (cutaneous and gastrointestinal) was associated with a lower probability of detecting specific *Firmicutes* taxa, including the *Ruminococcus gauvreauii* group, *Lachnospiraceae* NK4A136 group, and *Peptoclostridium*. No significant associations were identified in abundance-based models for either dietary treatment. Taxa summary containing median, minimum and maximum values can be found in Supplementary File S1.

## 4. Discussion

This study showed a clear clinical improvement in CE dogs, naïve to hydrolysed diets prior to enrolment, treated with a hydrolysed diet used as the first dietary intervention, with reductions in gastrointestinal signs, improvement in faecal score, and a decrease in CIBDAI. According to CIBDAI, all dogs improved in the present trial, whereas response rates reported in recent studies ranged from 60% to 87% [9,38,39]. Clinical improvement was observed within the first 30 days of both diets in 85% of dogs, while in the second period a shift toward stable or worsened status was noted, with no consistent additional improvement associated with *B. velezensis* supplementation compared with the hydrolysed diet alone. One dog showed a delayed improvement, with clinical response becoming evident only after two months, suggesting that delayed responses may occur in a small proportion of cases. Overall, our results support previous evidence indicating that dietary response, when present, is typically rapid [7,40]. Several studies have demonstrated the efficacy of hydrolysed protein diets in the management of CE in dogs. For example, a controlled, open-label, randomised trial comparing a hydrolysed soy protein and rice diet with a highly digestible diet containing intact proteins reported similar initial remission rates (88%) in both groups; however, most dogs in the control group subsequently relapsed, suggesting a delayed response to intact dietary proteins [7]. Similar findings were reported in a more recent study comparing a hydrolysed fish protein diet with rice starch, with or without prebiotics, with a limited-ingredient intact-protein diet (based on corn, fish, and chicken) [40]. Most dogs showed improvement in CCECAI scores within two weeks of treatment, regardless of the diet type. Dogs that did not respond to the initially assigned diet were then switched to the alternative dietary formulation, resulting in clinical improvement and further supporting the role of simplified ingredient profiles in the dietary management of CE [7,40]. In the present study, a non-hydrolysed control diet was not included. Nevertheless, the consistency of the clinical improvement observed with the hydrolysed diet confirms its usefulness as a therapeutic tool for veterinary clinicians. It should further be noted that at baseline, 62% of dogs had subnormal serum cobalamin levels and received oral or subcutaneous cobalamin supplementation throughout the study period. Cobalamin deficiency is a common finding in dogs with chronic enteropathy and has been associated with greater disease severity and a poorer prognosis [41]; consequently, its correction through supplementation is considered part of the standard therapeutic management of these patients, with reported benefits on clinical outcome, appetite, and body condition, as well as a potential role in modulating intestinal dysbiosis [42]. Therefore, it cannot be excluded that part of the clinical improvement observed over the study period was also influenced by cobalamin supplementation, in addition to the effects of the hydrolysed diet itself.

Another positive outcome was the maintenance or improvement of body weight and body condition score in most dogs, which are important clinical indicators in animals with chronic intestinal disease where maldigestion and malabsorption frequently lead to weight loss. A trend toward greater BCS improvement was observed at the end of the second dietary phase including *B. velezensis*, although this difference did not reach statistical significance. Among the studied dogs, three overweight animals were successfully managed through controlled feeding and returned to an ideal BCS on purpose. Overall, the ability to maintain or increase BW during the study suggests good digestibility and palatability of the hydrolysed diet, which likely contributed to the improvement of gastrointestinal signs. These findings are consistent with previous studies reporting moderate but significant BW increases in dogs receiving hydrolysed diets during the management of CIE [7].

The present study showed a positive outcome in the dietary management of canine CE but did not demonstrate a significant effect of *Bacillus velezensis* DSM 15544 on either clinical parameters or modulation of the intestinal microbiota. Except for one subject, all the other dogs enrolled in this study did not have abnormal DI or concentrations of core bacterial taxa.

An aspect worth considering is that clinical response in dogs with CE is not always directly associated with the degree of intestinal dysbiosis: previous studies have shown that dogs affected by food-responsive enteropathy (FRE) often exhibit only mild or even absent dysbiosis when assessed through microbiota-based indices, suggesting that clinical improvement following dietary intervention may occur independently of major microbiota shifts [43,44]. Conversely, dogs presenting with marked dysbiosis are generally less likely to respond satisfactorily to dietary treatment alone and may require additional therapeutic approaches [45,46].

In the present study, one dog exhibited a marked reduction in *Peptacetobacter* (*Clostridium*) *hiranonis*, a bacterial species involved in secondary bile acid metabolism and considered an important marker of intestinal microbial functionality [47,48]. Despite the persistence of this alteration during the study, the dog showed clear clinical improvement after dietary management. Moreover, the depletion of *P. hiranonis* has been suggested to reflect a functional disturbance of intestinal homeostasis and microbial bile acid metabolism that may not be readily reversible through dietary intervention alone [47,48]. Consequently, the absence of recovery of this bacterial taxon in the present study does not necessarily contradict the favourable clinical response observed following the dietary treatment.

Interestingly, some dogs receiving the probiotic showed a temporary worsening of diarrhoea, and 31% developed mild ocular and periocular signs, including epiphora and conjunctival hyperaemia. These findings could reflect individual intolerance to the probiotic strain or sensitivity to residual components of the culture medium used for its production. Notably, the probiotic strain used in this study, *Bacillus velezensis* DSM 15544, is authorised by the European Food Safety Authority (EFSA) as a feed additive for dogs, and its original safety assessment specifically excluded dermal and ocular irritant potential [31]; this would argue against a direct irritant effect of the strain itself, although residual carrier or fermentation-derived components were not independently assessed. However, the relationship between such signs and food-related reactions in dogs remains poorly documented, and no evidence currently links probiotic supplementation with ocular manifestations [49,50]. Therefore, this hypothesis remains speculative [51,52].

The literature on probiotic treatments in CE dogs remains limited, and comparisons with current results are challenging because probiotic strains, dosages, and routes of administration vary considerably across studies. To date, most available research on CE has focused on a restricted number of probiotic formulations, including single-strain or multi-strain lactic acid bacteria (LAB) mixtures [19], *Enterococcus faecium* NCIMB 10415 [22,53], and *Saccharomyces boulardii* [20]. Single and multi-strain LAB preparations, together with *S. boulardii*, seem to provide the strongest evidence for immunoregulatory and barrier-supporting effects [19,20,21], whereas the clinical benefit of *E. faecium* NCIMB 10415 in dogs with FRE appears limited [22,53].

About *B. velezensis*, to the authors’ knowledge, only two randomised, double-blind, controlled studies have been conducted in dogs with enteropathies. In the first study, the probiotic was administered together with a hydrolysed diet, either alone or in combination with *Ascophyllum nodosum* (a macroalga rich in soluble fibre and gums), and it was provided in encapsulated form at a relatively high dosage (12.5 × 10^9^ CFU per kg of BW) [54]. Although no significant clinical improvement was observed, treated dogs showed higher microbial abundance and increased concentrations of volatile fatty acids, including acetate, isovalerate and isobutyrate, as well as a trend toward increased butyrate compared with controls [54]. A more recent study, conducted in dogs with acute enteritis, evaluated the probiotic applied as a coating on the kibble, similarly to the approach used in the present study, but using diets based on non-hydrolysed proteins [25]. The control diet was also based on non-hydrolysed chicken protein, but differed in ingredient composition, containing lower amounts of corn gluten, rice, and corn, and lacking the additional components present in the test diet (FOS, sepiolite, and coconut oil), while including beet pulp as a fibre source. Differences were also present in macronutrient composition; although overall digestibility was reported to be similar, the control diet contained higher levels of fat and crude fibre. The significantly faster recovery of gastrointestinal signs and improvement in microbiota quality observed with the fortified diet may therefore not be exclusively attributable to probiotic supplementation, as also acknowledged by the authors [25]. Furthermore, although the probiotic was reported to remain stable on the kibble for up to 18 months, no analytical data on probiotic recovery or shelf-life stability in the test diet were provided.

Studies investigating other probiotic formulations administered with similar protocols have also produced inconsistent results. For example, the administration of a symbiotic preparation containing *Enterococcus faecium* NCIMB 10415 (E1707) at a concentration of 1 × 10^9^ CFU combined with fructo-oligosaccharides and arabic gum, given once daily irrespective of BW together with a hydrolysed soy protein and rice diet, did not demonstrate a clear advantage compared with the control group; however, the number of enrolled dogs was insufficient to draw definitive conclusions [22]. Similarly, the administration of three *Lactobacillus* strains (*L. acidophilus* NCC2628 and NCC2766, and *L. johnsonii* NCC2767) at a concentration of 1 × 10^10^ CFU per each strain per gram of supplement, given at a daily dose of 1 g for one month regardless of BW, resulted in only modest clinical improvement when combined with a fish (salmon and trout), canola meal and rice based diet [55]. In that study, similar to our findings, the clinical benefit appeared mainly attributable to the dietary intervention rather than to the probiotic supplementation, and only *L. johnsonii* NCC2767 was detected in the faecal samples of some dogs [55]. Overall, positive effects of probiotics in canine CE appear more likely when higher dosages are administered or when different strains or strain combinations are used. In several studies reporting beneficial outcomes in CE, probiotics were administered independently of the diet and often at substantially higher daily doses than those achievable through feed coating technologies. Furthermore, many of those trials included dogs refractory to dietary treatment, which may represent a different clinical population compared with the animals enrolled in the present study. Indeed, in our study, all dogs showed clinical improvement following the hydrolysed diet, which may have obscured any additional beneficial effects attributable to the probiotic strain. A study evaluated the probiotic potential of *Saccharomyces boulardii* in dogs with CE [20]; when it was administered in encapsulated form at a dosage of 1 × 10^9^ CFU/kg of BW to both healthy and CE-affected dogs, treated animals showed a significant improvement in CCECAI scores compared with the placebo group. The encapsulated administration allowed precise dosing and protection of probiotic viability during gastrointestinal transit, and the dosage based on the actual BW of animals guaranteed a much higher amount compared to our study.

A potential limitation of the present trial concerns the relatively small sample size. Nonetheless, the sample size adopted (*n* = 12) is consistent with those employed in previous trials investigating *Bacillus subtilis*-based probiotics in dogs, which have generally included between 8 and 12 animals [27,29,56,57]. Another limitation is represented by the absence of a washout period between the two dietary phases and by the lack of a microbiological baseline comparable to the pre-switch time point. Although this aspect was accounted for in the statistical model, a possible residual influence of the first treatment period on the second cannot be entirely excluded and the data presented here should be interpreted with caution.

Finally, the mode of probiotic administration, which was itself one of the aspects investigated, should also be considered. In this study, in fact, the probiotic was applied as a coating on the diet, resulting in an intake proportional to food consumption. A previous investigation suggests that body weight explains differences in microbiota composition better than chance, but not overwhelmingly [58]. This approach may lead to relatively low probiotic exposure in small dogs in this study, whereas large dogs consuming greater quantities of food may receive amounts closer to those used in other studies, where probiotics were given encapsulated. The administration of encapsulated, rather than coated, probiotics allowed for precise dosing and protection of probiotic viability during gastrointestinal transit. The effective dose and viability of spores depend on multiple aspects, including the feeding amount, the initial probiotic concentration, processing conditions, application technology, and product shelf life. The registered dosage for intestinal stabilisation is 1 × 10^9^ CFU/kg of feed. Although the recovered concentration in the present study reached approximately 80% of the recommended level, this amount may still have been insufficient to exert measurable microbiological effects.

## 5. Conclusions

Dogs showed overall clinical improvement during the study, with the hydrolysed diet supporting improvement in clinical signs. Supplementation with *Bacillus velezensis* via post-extrusion coating was well tolerated by most dogs; however, the marked clinical improvement associated with the hydrolysed diet may have masked any additional clinical benefits attributable to the probiotic. These findings highlight the effectiveness of dietary management in canine chronic enteropathy and indicate that probiotics can be safely integrated as a complementary nutritional approach. Overall, while *B. velezensis* DSM 15544 exhibits characteristics compatible with a functional probiotic candidate in canine nutrition, its clinical efficacy in the context of chronic enteropathy remains to be fully elucidated, warranting further controlled investigations.

## Figures and Tables

**Table 1:** Composition of the extruded dog food used for the dietary trial.

Nutrients	g/100 g, as Fed	g/Mcal ME
DM	93.7	
CP	26.6	87
EE	8.3	27
Ash	8.7	28
CF	1.6	5.2
NFE	40.4	131
TDF	9.7	32
SDF	2.5	8.2
IDF	7.2	23.8

DM: dry matter; CP: crude protein; EE: ether extract; CF: crude fibre; IDF: insoluble dietary fibre; NFE: nitrogen-free extract; SDF: soluble dietary fibre; TDF: total dietary fibre; Data are expressed as the mean value based on the analysis of the two diet batches (DIET and DIET+PRO). ME: metabolisable energy calculated using modified Atwater factors: 305 kcal/100 g. Composition (% as fed basis): Hydrolysed fish (herring) (34%), rice, potato starch, coconut oil (6%), fish oil (herring), sunflower oil, chicory root (1%), psyllium (1%), inactivated yeasts (*S. cerevisiae*, *C. jadinii,* 1%), xylo-oligosaccharides (0.3%), yucca juice.

**Table 2:** Body weight (BW) and body condition score (BCS) in 10 study dogs during the two dietary interventions (DIET and DIET+PRO), at each time point (T1 and T2). Among all the enrolled dogs (*n* = 13), 3 subjects were excluded from the analysis as they underwent a weight loss regimen. Data are expressed as mean ± SD.

		DIET+PRO		DIET		*p*-Value		
	T0	T1	T2	T1	T2	Diet	Time	Diet x Time
BW	15.1 ± 10.3	15.3 ± 10.2	15.4 ± 10.1	15.2 ± 10.2	15.1 ± 10.0	0.75	0.67	0.67
BCS	3.8 ± 0.6	4.0 ± 0.7	4.3 ± 0.8 *	3.8 ± 0.6	3.9 ± 0.6	0.27	0.21	0.21

* *p*-value = 0.05 when T2 was compared to T0.

**Table 3:** Faecal pH, NH_3_, IgA and volatile fatty acids (total VFA and % of total VFA) in the study dogs during the two dietary interventions (DIET and DIET+PRO) at each time point (T1 and T2). Data are available for 13 dogs at T0 and T1, and for 12 dogs at T2, as one dog did not complete the study. Data are expressed as mean ± SD.

	DIET+PRO		DIET		*p*-Value		
	T1	T2	T1	T2	Diet	Time	Diet x Time
pH	7.3 ± 0.2	7.2 ± 0.2	7.2 ± 0.5	7.2 ± 0.4	0.82	0.21	0.96
NH_3_ (µmol/g as is)	50.1 ± 29.9	44.2 ± 9.6	46.4 ± 19.6	42.4 ± 13.8	0.82	0.57	0.94
IgA (mg/g DM)	4.9 ± 4.2	8.7 ± 7.6	6.9 ± 6.1	5.3± 3.6	0.28	0.67	0.96
Total VFA (µmol/g as is)	83.2 ± 25	80.6 ± 16.8	75.6 ± 24.2	79.8 ± 20.5	0.80	0.31	0.96
Acetate (%)	55.1 ± 7.2	55.8 ± 5.6	57.4 ± 7.4	56.0 ± 7.0	0.56	0.37	0.38
Propionate (%)	30.0 ± 6.4	29.2 ± 5.2	28.3 ± 6.4	28.3 ± 7.6	0.29	0.19	0.33
Isobutyrate (%)	2.0 ± 0.8	1.9 ± 1	2.1 ± 1.7	1.9 ± 1.1	0.95	0.98	0.66
Norbutyrate (%)	10.1 ± 2.4	10.5 ± 2.5	9.5 ± 3.2	11.4 ± 3.9	0.21	0.28	0.44
Isovalerate (%)	2.7 ± 1.3	2.6 ± 1.5	2.7 ± 2.5	2.4 ± 1.6	0.95	0.96	0.66

VFA: volatile fatty acid.

**Table 4:** Changes in prevalence and abundance of different taxa identified by the MaAsLin3 comparing the two interventions (DIET and DIET+PRO).

Taxonomic Level	Coeff	SE	*p*-Value	q-Value	Model
p. Actinobacteroidota	2.918	0.667	0.041	0.987	abundance
c. Coriobacteriia					
o. Coriobacteriales					
f. Eggerthellaceae					
*g. Eggerthella*					
p. Firmicutes	4.331	2.067	0.036	0.991	prevalence
c. Bacilli					
o. Bacillales					
f. Bacillaceae	4.174	2.068	0.044	0.987	prevalence
* * *g. Bacillus*					
o. Lactobacillales	1.807	0	0.041	0.987	abundance
f. Carnobacteriaceae					
f. Lactobacillaceae	−5.397	1.726	0.032	0.993	abundance
* * *g. Lactobacillus*					
c. Clostridia	−1.459	0.659	0.027	0.987	prevalence
o. Oscillospirales					
f. Ruminococcaceae					
* * *g. Fournierella*					
o. Peptostreptococcales	1.409	0.824	0.045	0.987	abundance
f. Peptostreptococcaceae					
* * *g. Romboutsia*					

p., phylum; c., class; o., order; f., family; g., genus. Coefficient = effect size; SE = standard error; *q*-value = FDR-adjusted *p*-value; model = prevalence/abundance (MaAsLin3).

## Data Availability

The raw data supporting the conclusions of this article will be made available by the authors on request.

## Supplementary Materials

The following supporting information can be downloaded at https://www.mdpi.com/article/10.3390/ani16162581/s1.

## Abbreviations

The following abbreviations are used in this manuscript:

ASVs	Amplicon sequence variants
BCS	Body condition score
BW	Body weight
CBC	Complete blood count
CFU	Colony-forming unit
CE	Chronic enteropathy
CF	Crude fibre
CP	Crude protein
CIBDAI	Canine Inflammatory Bowel Disease Activity Index
DI	Dysbiosis Index
DIET	Treatment with the hydrolysed diet alone
DM	Dry matter
DIET+PRO	Treatment with the hydrolysed diet supplemented with the probiotic strain *Bacillus velezensis* DSM 15544
EE	Ether extracts
EFSA	European Food Safety Authority
FOS	Fructooligosaccharide
FREs	Food-responsive enteropathies
FS	Faecal score
GALT	Gut-associated lymphoid tissue
GLM	Generalised linear model
IDF	Insoluble dietary fibre
IgA	Immunoglobulin A
LAB	Lactic acid bacteria
MaAsLin3	Multivariable Association with Linear Models 3
ME	Metabolisable energy
NFE	Nitrogen-free extract
SD	Standard deviation
SDF	Soluble dietary fibre
PERMANOVA	Permutational multivariate analysis of variance
TDF	Total dietary fibre
T0	Baseline of the study (enrolment)
T1	Sampling time point, corresponding to 30 days of dietary trial
T2	Sampling time point, corresponding to 60 days of dietary trial
UPC	Protein-to-creatinine ratio
VFAs	Faecal volatile fatty acids
