# Supplementary material for: Clinical and Faecal Outcomes in Adult Dogs with Chronic Enteropathy Fed an Hydrolysed Protein Diet with and Without Bacillus velezensis DSM 15544: A Randomised, Double-Blind, Cross-Over Trial
Source: Animals (Basel). 2026 Aug 18;16(16):2581. doi: 10.3390/ani16162581 (PMC13508852; doi:10.3390/ani16162581)
Supplement: Supplementary file 1 [file animals-16-02581-s001.zip › Beta_diversity_adverse_effect_SFigure2.pdf]

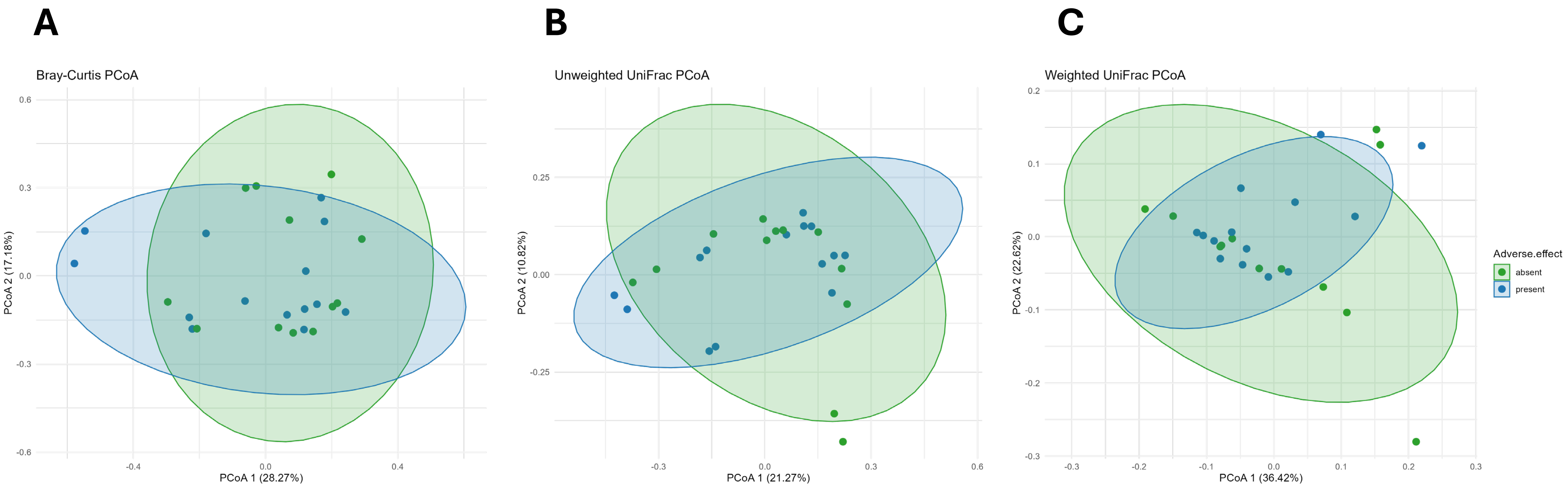
A
Bray-Curtis PCoA
PCoA 2 (17.18%)
PCoA 1 (28.27%)
0.6
0.3
0.0
-0.3
-0.6
-0.4
0.0
0.4
B
Unweighted UniFrac PCoA
PCoA 2 (10.82%)
PCoA 1 (21.27%)
0.25
0.00
-0.25
-0.3
0.0
0.3
0.6
C
Weighted UniFrac PCoA
PCoA 2 (22.62%)
PCoA 1 (36.42%)
0.2
0.1
0.0
-0.1
-0.2
-0.3
-0.3
-0.2
-0.1
0.0
0.1
0.2
0.3
Adverse.effect
absent
present
