# Supplementary material for: Clinical and Faecal Outcomes in Adult Dogs with Chronic Enteropathy Fed an Hydrolysed Protein Diet with and Without Bacillus velezensis DSM 15544: A Randomised, Double-Blind, Cross-Over Trial
Source: Animals (Basel). 2026 Aug 18;16(16):2581. doi: 10.3390/ani16162581 (PMC13508852; doi:10.3390/ani16162581)
Supplement: Supplementary file 1 [file animals-16-02581-s001.zip › Beta_diversity_all_groups_SFigure1.pdf]

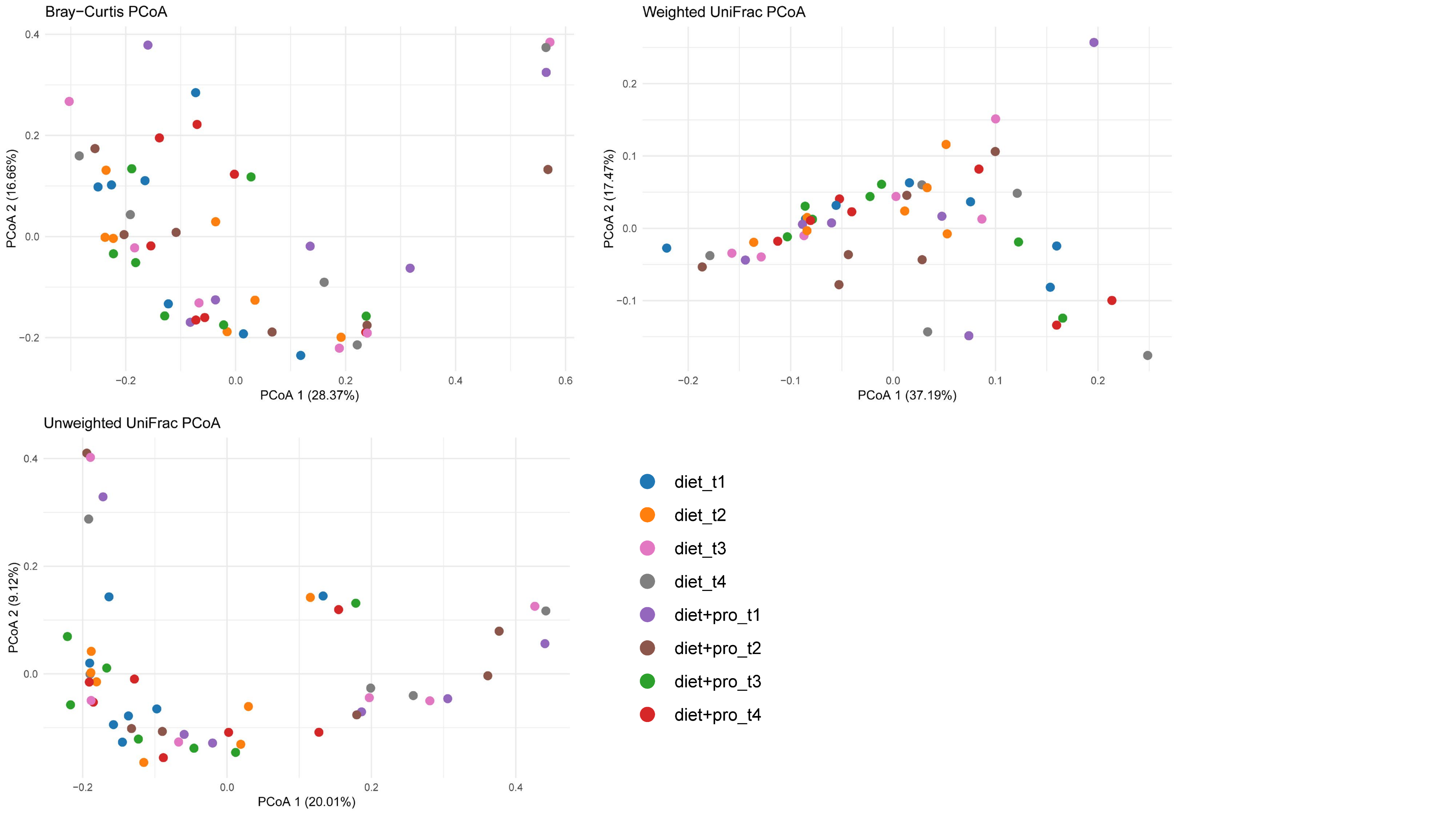
Bray−Curtis PCoA
PCoA 1 (28.37%)
PCoA 2 (16.66%)
Weighted UniFrac PCoA
PCoA 1 (37.19%)
PCoA 2 (17.47%)
Unweighted UniFrac PCoA
PCoA 1 (20.01%)
PCoA 2 (9.12%)
diet_t1
diet_t2
diet_t3
diet_t4
diet+pro_t1
diet+pro_t2
diet+pro_t3
diet+pro_t4
